# Influence of the Filler Particles’ Surface Morphology on the Polyurethane Matrix’s Structure Formation in the Composite

**DOI:** 10.3390/polym13223864

**Published:** 2021-11-09

**Authors:** Taisiya A. Shalygina, Mikhail S. Rudenko, Ivan V. Nemtsev, Vladimir A. Parfenov, Svetlana Y. Voronina, Igor D. Simonov-Emelyanov, Polina E. Borisova

**Affiliations:** 1 Smart Materials and Structures Lab., Department of Aircraft, Reshetnev Siberian State University of Science and Technology, 31 KrasnoyarskyRabochy Av., Krasnoyarsk 660037, Russia; mister.m.rudenko@gmail.com (M.S.R.); simkina_svetlana@mail.ru (S.Y.V.); polina.nabieva.97@mail.ru (P.E.B.); 2 L.V. Kirensky Institute of Physics SB RAS, Institute of Chemistry and Chemical Technology, Federal Research Center “Krasnoyarsk Science Center of the Siberian Branch of the Russian Academy of Sciences”, 50 Akademgorodok, Krasnoyarsk 660036, Russia; ivan_nemtsev@mail.ru (I.V.N.); parf-va@mail.ru (V.A.P.); 3 Lomonosov Institute of Fine Chemical Technologies, MIREA—Russian Technological University, Vernadskogo Avenue 86, Moscow 119571, Russia; simonov@mitht.ru

**Keywords:** polyurethane, surface morphology, nanoparticles, nanowires, core-shell, molecular mobility, molecular heterogeneity, interfacial layers, boundary layer, transition layer

## Abstract

This article presents the surface morphology effect of silicon carbide (SiC) particles on the polyurethane binder’s structure formation in a dispersed-filled composite. The difference in the morphology and surface relief of filler particles was ensured by the implementation of plasma chemical modification. As a result of this modification, the filler consisted of core-shell particles characterized by a SiC core and a carbon shell (SiC@C), as well as a carbon shell decorated with silicon nanoparticles (SiC@C/SiNP) or nanos (SiC@C/SiNW). The study of the relaxation properties of polyurethane composites has shown that the strongest limiting effect on the molecular mobility of boundary layer’s chain segments is exerted by a highly developed surface with a complex relief of SiC@C/SiNP and SiC@C/SiNW particles. An empirical method was proposed to find the polymer fractions spent on the formation of the boundary, transition and bulk layers of the polymer matrix in the composite. It was shown that the morphology of the filler particles’ surface does not affect the dependence of the boundary layer thickness on the filler’s volume fraction. However, with an increase in the degree of surface development, the boundary layer thickness decreases.

## 1. Introduction

Polymer composite materials are still considered to be an actively developing area of modern materials science. They are used in various fields of science and technology: medicine, aviation and automotive industry, electronics, food processing industries, etc. [1,2]. Reinforcement and filling with dispersed fillers can significantly expand the application’s scope, due to the new operational and functional properties acquisition [3,4,5,6]. Due to the fact that Polymer Composite Materials (PCM) do not obey the mixture rule, an accurate prediction of their potentially wide-ranging properties is very difficult.

The difficulties lie in the fact that the properties of only two phases are taken into account when modeling the final properties of PCM: the polymer matrix and the filler. Therefore, theoretical calculations often do not coincide with experimental data [7,8,9,10]. The article [11] shows that the polymer’s matrix phase in the composite should be considered as the sum of the three constituent elements, all of which have their own specific role in the matrix structure’s organization. One of the constituent elements is the boundary layer, which is in direct contact with the filler surface. Moreover, a transition layer with a looser packing of polymer chains can form between the boundary layer and the polymer’s layer. The structure and properties of these layers will differ in many ways from the polymer properties in volume, and therefore can contribute to the final composite’s properties [12]. Obtaining each layer’s results separately during the experiment is both crucial and difficult [13].

The polymer chains complex organization within the composite is characterized by the microheterogeneous–system, the degree of which depends on many parameters: the chains’ chemical structure, the polymer with the filler surface’s adsorption interaction, the composite manufacturing conditions (polymerization in the presence of filler, filling of polymer melt), etc. [14].

A real idea of the structure formation and boundary’s layers properties was formed on the basis of the results of thin polymer films on various substrate studies [15,16]. However, by revising the composites obtained by polymerization in a filler surface’s presence, a somewhat different picture is observed.

Until recently, it was believed that the boundary layer’s structure formation is mainly affected by the polymer’s interaction with the surface filler [17,18]. The filler’s plasticizing effect is observed in the absence of any interactions at the interface. To ensure the most effective interaction at the polymer/filler interface, the filler surface requires careful evaluation using suitable analysis methods [19]. The formation of covalent or non-covalent bonds between the polymer and the filler leads to the fact that the filler surface has a limiting effect on the polymer chain segment’s molecular mobility, due to their conformational set’s depletion. In this case, there is an increase in the glass transition temperature (Tg) and an improvement in physical and mechanical properties [20]. In order to enhance interactions at the polymer/filler interface, various covalent and non-covalent functionalization methods are used to promote the formation of active functional groups on the filler surface [21]. A fairly complete description of surface functionalization methods suitable for carbon fillers can be found in the review [22]; in addition, one can find the use of other techniques that allow for interfacial interaction. Such methods include polymerization of the polymer in situ on the filler surface [23] or the use of halloysite nanotubes (HNT), the aluminol and siloxane groups on the surface of HNT, which facilitate the formation of hydrogen bonding with the biomaterials onto its surface as a filler [24,25].

However, the results of computer modelling of dispersed-filled polymer composites’ structure have shown that, along with any interaction of polymer chains with the dispersed filler’s surface, the morphology (relief) of this surface should be taken into account [26]. They showed a significant difference in the variation in parameters such as polymer density in the boundary layers and the amount of free volume in the interfacial region, depending on whether the particle has a smooth surface or a bristle-like relief. Currently, there is a lack of statistical experimentally obtained results that allow us to assess the effect of the filler particles’ surface morphology on the boundary layer’s structure formation, the degree’s microheterogenicity, composites’ relaxation and functional properties.

Therefore, this work is devoted to the study of the influence of silicon carbide (SiC) particles’ surface morphology on a polyurethane composite’s structure formation and properties. The choice of SiC is due not only to its unique properties, which allow for the increase in thermal conductivity and the modulus of the composite’s elasticity, but also to the physical modification of the particles’ possibility, which allows for the control of their surface’s morphology (relief) to assess its effect on the polyurethane matrix’s structure formation. We have developed a method of SiC particles’ plasma chemical modification. During the modification process, core-shell particles characterized by a SiC core and a carbon shell (SiC@C), and a carbon shell decorated with silicon nanoparticles (SiC@C/SiNP) or (SiC@C/SiNW), were obtained.

## 2. Materials and Methods

### 2.1. Materials

#### 2.1.1. Polymer Matrix

The polymer matrix was a reactoplastic polyurethane binder (PU) based on ε-polycaprolactone (PCL, 99%)—component B (Viscosity 200–600 MPa·s) and aromatic diisocyanate (MDI, 98%)—component A (Viscosity 200–800 MPa·s) of the Diaplex MP5510 brand (Japan).

#### 2.1.2. Filler

**Figure 1:**
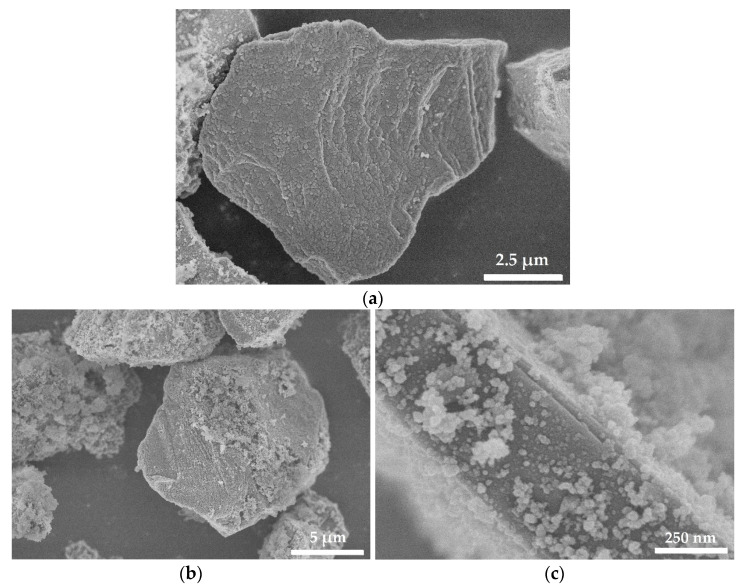

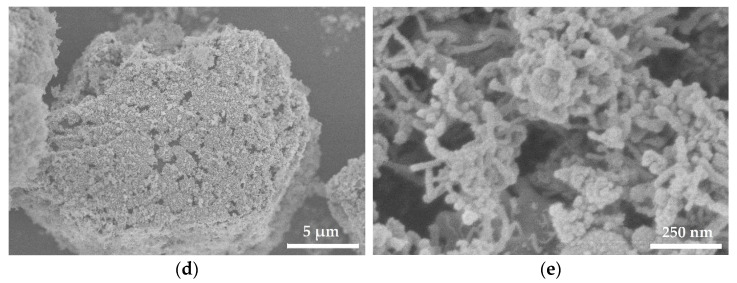
Scanning electron microscopy (SEM) image of modified particles SiC@C (**a**), SiC@C/NP (**b**,**c**), SiC@C/NW (**d**,**e**).

The initial (purity 98%) and plasma-chemically modified SiC particles of quasi-spherical shape with a particle diameter from 6 to 10 μm were used as a filler. The filler particles’ introduced designations were: initial—SiC_neat, modified SiC particles according to their surface morphology, SiC@C—SiC particles with a carbon shell, SiC@C/SiNP—SiC particles with a carbon shell decorated with silicon nanoparticles (SiNP), SiC@C/SiNW—SiC particles with a carbon shell decorated with silicon deposits (SiNW). The morphological features of the powder were visualized using a high-resolution scanning electron microscope FE-SEM S-5500 (Hitachi Ltd., Tokyo, Japan) with an Accelerating Voltage of 3 kV, a current of 20 pA, Magnification k5x, Working Distance 0.2 mm. All images were taken on FE-SEM in secondary electrons (SE) with a maximum resolution of 1920 × 1920 px (5 MP) for deeper detail and the slowest scan (scanmode) to minimize “noise”. The diameter of SiNP ranged from 30 to 60 nm, SiNW were characterized by a diameter of about 50 nm, a length of about 400 nm (Figure 1). The values of the SiC particles’ specific surface area are presented in Table 1.

### 2.2. Sample Preparation

In the composite’s manufacture, the filler was introduced into the component in PU, after which it was mechanically dispersed using a top-drive agitator for 30 min at 1000 rpm. After that, component A was added to the ratio (60/40), mixed and evacuated for 5 min. Next, the composition was poured into an aluminum tooling, the geometry of which controlled the specified thickness of the samples (0.7 mm). Composite polymerization was carried out in a climatic chamber at 80 °C for 4 h.

The objects of the study were initial polyurethane samples (PU_neat), polyurethane filled with initial SiC particles (PU/SiC_neat) and polyurethane filled with modified SiC particles (PU/SiC@C, PU/SiC@C/SiNP and PU/SiC@C/SiNW).

### 2.3. Measurements

#### Dynamic Mechanical Analysis (DMA)

The composite relaxation properties were captured through dynamic mechanical analysis (DMA), using a dynamic mechanical analyzer (DMAQ800, manufactured by TAInstruments, USA). The samples were analyzed using the shear deformation mode with a frequency of 1 Hz, a constant amplitude of 15 μm, in the temperature range from 15 to 150 °C. The samples were made in the plates measuring 10 × 10 mm and 0.7 mm thick form.

## 3. Results and Discussion

### 3.1. Dependence of the Composite’s Relaxation Properties from the Filler Particle Surface Morphology and Its Concentration

Studies of the polyurethane composite samples’ relaxation properties were carried out in a wide range of the filler’s volume fraction values for the possibility of assessing the structure of the polyurethane binder’s microheterogenicity. The determination of the composites’ relaxation properties at a high degree of filling makes it possible to estimate the chain segments forming the boundary layer’s mobility. For this purpose, the maximum filler fraction (φ*_m_*) was determined experimentally, which defined the maximum possible filler content in the composite while maintaining its monolithicness. Therefore, the filler concentration range’s boundary value (φ*_f_*) should be close to φ*_m_*.

As you can see from Table 1, there was a decrease in the φ*_m_* values for particles with a brush-like surface, which could contribute to the achievement of the required final properties at significantly lower filler concentrations. According to article [27], the relationship between the type of dispersed-filled composites’ spatial lattice and the filler particles’ shape, size and specific surface area was determined. According to this classification, unmodified SiC_neat particles corresponded to filler microparticles with a size from 1 to 10 μm and φ*_m_* from 0.255 to 0.45 vol. d., which meant they formed a tetrahedral lattice with a coordination number Z = 4. An increase in the specific surface area of particles with a brush-like surface (SiC@C/NP and SiC@C/NW) led to a change in the coordination number (Z = 3) of the tetrahedral spatial lattice in the composite.

Considering the thesis within materials science that structure determines properties, the structure should be described only in volume units. It cannot be represented in mass units, because, in this case, the ratio of the initial components’ densities should be taken into account [28]. Therefore, to calculate the filler’s volume fractions and select a suitable concentration range, a ratio describing the relationship between the volume and mass units of the filler content (φ*_fv._* and φ*_fw._*) in a two-phase composite was used [29]:

φ*_fv_* = (ρ/ρ_p_)/[(1/φ_fw_) + (ρ/ρ_p_) − 1], (1)

where ρ*_p_* is the filler’s bulk density; ρ is filler’s true density.

Table 2 shows the values of the weight concentrations’ selected range and the corresponding values of the filler’s volume fractions (φ*_f_*).

**Figure 2:**
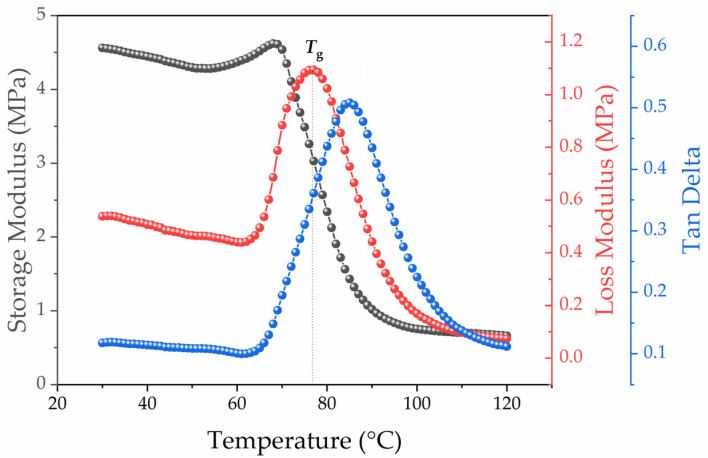
DMA of unfilled polyurethane sample curves.

Figure 2 shows typical DMA–curves of the initial polyurethane’s cured sample, characterized by the temperature dependence of the shear modulus *G*, the mechanical loss modulus *G* and the tangent of the mechanical loss angle (tan δ). The temperature value of the mechanical loss module’s maximum peak was chosen for the glass transition temperature (*T_g_*). Each peak maximum *G*″(*T*) characterizes a new type of defrosting of polymer chains’ segmental movement.

**Figure 3:**
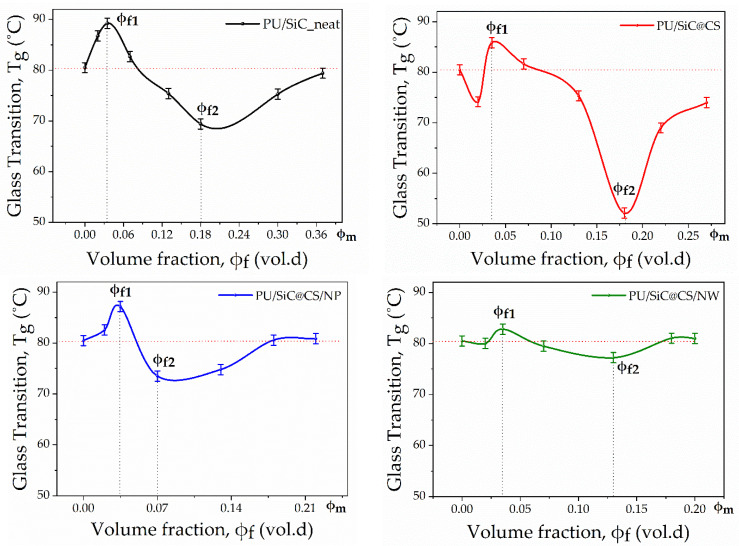
The glass transition temperature *T_g_* dependence on the concentration and type of morphology of the filler particles’ surface.

The DMA curves presented above were obtained for all samples of polyurethane binder filled with initial and modified SiC particles with controlled surface morphology in the concentration range according to the values given in Table 2. Figure 3 shows the glass transition temperature’s dependence on the filler’s volume fraction and the type of surface morphology. During the statistical analysis of the data from three samples, the average spread of values in the sample was determined, having a value less than the value of the error of the thermocouple. Therefore, Figure 3 shows the error of the device in temperature, which was ±1 °C. As you can see in Figure 3, this dependence was non-monotonic, the minimum lay in the area corresponding to 50% of the system’s filling. The non-monotonic nature of the concentration dependence of the composites’ glass transition temperature confirmed the high degree of microheterogenicity in the polyurethane matrix’s structure. The change in the *T_g_* composite’s value, dependent on the degree of filling, indicated the complex organization of the binder’s polymer chains into a heterogeneous structure. This structure consisted of boundary, transition and bulk layers, all of which were characterized by its own chain segment’s mobility value and *T_g_*. The obtained *T_g_* values shown on the graphs characterize the *T_g_* values’ superposition of each structural layer. It can be concluded that the total *T_g_* value of the entire system at a certain filler concentration is the result of the dominance of a binder’s particular structural layer. The noted nonmonotnic changes in the relaxation properties of composites may also be related to the nonmonotonic changes in the structure of polymer boundary layers on the filler particles’ surface when their volume fraction in the composite changes [30].

Moreover, it is important to note that the minimum range of variation in *T_g_* values from the filler concentration was populated by samples of polyurethane composite filled with modified SiC@C/NP and SiC@C/NW particles. This indicated that an increase in the SiC microparticles’ specific surface area led to a decrease in the degree of the polymer binder’s microheterogenicity in the composite. It can be assumed that, with an increase in the filler particles’ specific surface area, the boundary layer thickness decreases, while the complex relief of the filler particles’ surface can contribute to the overlapping area’s formation with interpenetrating networks of contacting layers.

Furthermore, we would highlight that the glass transition temperature was at a high degree of the composite’s (φ*_f_* = φ*_m_*) filling, which reflected the molecular mobility of polyurethane chains at the interface of the polymer with the filler particles’ surface. At a concentration close to the filler’s maximum volume fraction, the polymer layer between the filler particles was thinned and the polymer binder passed into the state of the boundary layer. Thus, at a concentration close to φ*_m_*, an increase in *T_g_* was observed, which exceeded the *T_g_* value of the initial polyurethane binder, only in the case of SiC@C/NP and SiC@C/NW particles’ filling. This means that the polyurethane chains’ mobility in the boundary layer was severely limited by the filler surface with a more complex relief.

The obtained results were in agreement with the data published in the above-mentioned article [26], in which the authors showed that, for composites filled with particles with a brush-like relief and an attractive surface, the polymer layer of the binder was compacted at their surface. Subsequently, the polymer density decreased, but returned to the bulk density just beyond the area of the brush-like relief.

### 3.2. Determination of the Polymer’s Proportion Forming the Boundary, Transition and Bulk Matrix Layer

We have proposed an empirical method for studying the polymer matrix’s structure, finding the fraction of the polymer forming the boundary (φ*_b_*), transition (φ*_t_*) and volume (φ*_v_*) layers.

**Figure 4:**
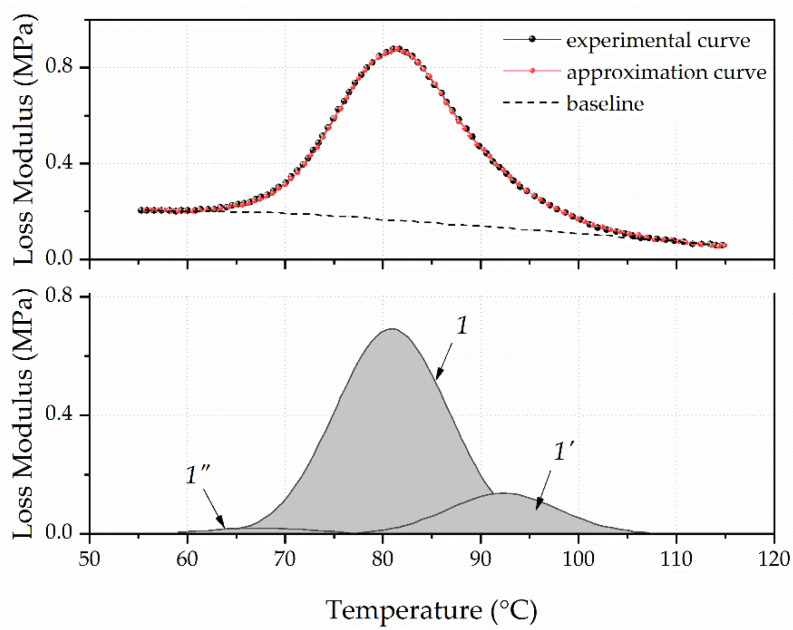
Approximation of the experimental peak of the unfilled polyurethane’s mechanical loss modulus.

The asymmetric and wide peak of the mechanical loss modulus on temperature dependence for unfilled polyurethane was a superposition of peaks (Figure 4). They reflected the temperature transition to a flexible segments’ highly elastic state that contained an ester group and rigid segments, which contained a urethane group characterized by a different molecular mobility. It was shown in articles [31,32] that the asymmetric peaks of *E*″ or tan(δ) must be graphically decomposed to identify all types of polymer chain segmental movement that made up a common peak.

Such decomposition into components of the dependence’s *G*″(*T*) peak obtained for unfilled polyurethane was performed using the PeakFit software. The baseline’s position was approximated by a linear function (Leaner (D2)), and the elementary peaks’ shape was set by the Gauss Amp. Moreover, the decomposition of the dependent *G*″(*T*) peak was carried out with knowledge of the polyurethane’s stoichiometric composition, and accounted for the fact that one urethane group accounted for six ester groups. Thus, the area of the dependent *G*″(*T*) peak characterized the temperature transition to the highly elastic state of rigid segments should have been 16% of the total peak area. 

The upper part of Figure 4 shows a dashed line, which represents the dependent *G*″(*T*) experimental curve. The solid line represents the curve obtained as a result of approximation. In the graph’s lower part, a peak with a maximum of 92 °C and a relative area of 16.07% characterizes the rigid segments’ relaxation properties. A peak with a maximum of 67 °C and a relative area of 2.22% characterizes the flexible segments’ relaxation properties with increased molecular mobility. This mainly includes segments located at the ends of the chain that are not involved in intermolecular binding. The peak with a maximum of 81 °C and a relative area of 81.7% is due to the temperature transition to a highly elastic state of polyurethane chains’ flexible segments.

**Figure 5:**
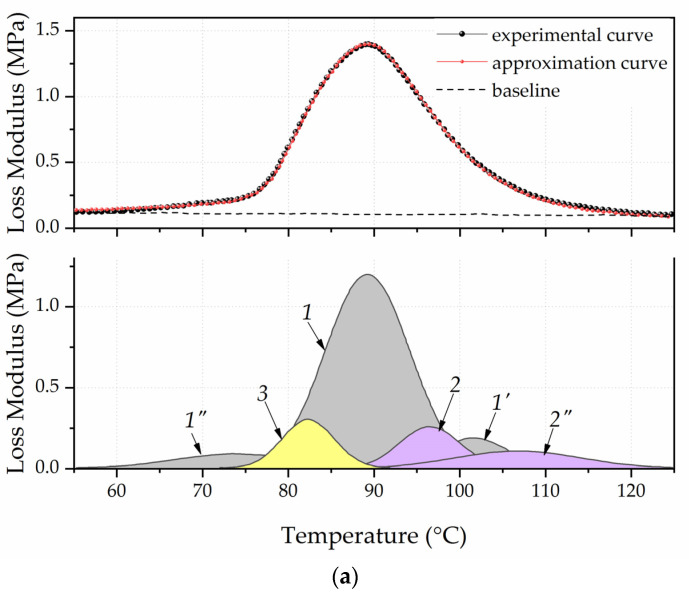

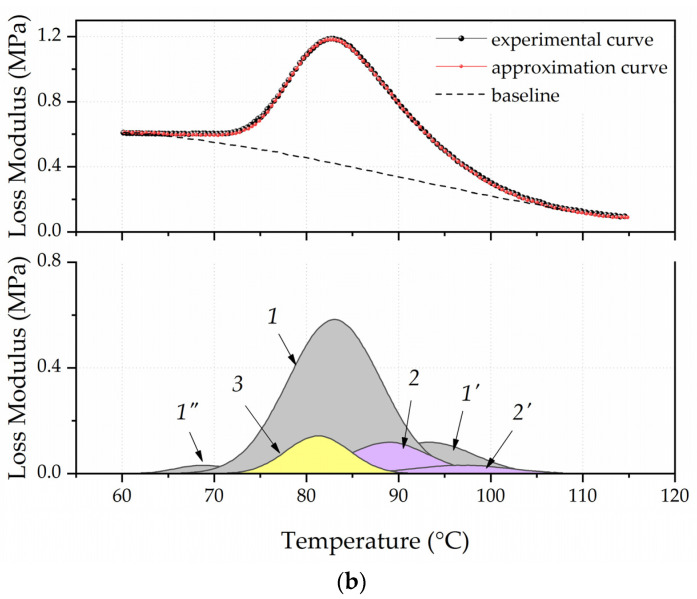
Approximation of the experimental peak of the polyurethane’s mechanical loss modulus filled with 0.035 vol. d particles SiC_neat (**a**) and SiC@C/NW (**b**).

Figure 5 shows the spectra’s decomposition of the polyurethane’s mechanical loss modulus filled with SiC_neat and SiC@C/NW particles (φ*_f_* = 0.035 vol. d.).

During the decomposition peaks, it was important to adhere to strict approximation conditions: the peaks total relative area characterized the temperature transition to a highly elastic state of rigid segments that made up a particular layer, and should be 16%. The value of the full width at half amplitude (full width at half maximum) (FWHM) should be close to the FWHM of peaks obtained by decomposing the curve of unfilled polyurethane.

The decomposition peaks are shown in the lower part of the graph, while the peaks that characterized the polyurethane chains’ proportion were not affected by the field of filler’s surface forces. As such, they are a part of the bulk layer of the matrix and are shaded in gray (1, 1′, 1″). The polymer’s total fraction forming the bulk layer (φ*_v_*) for the PU/SiC_neat and PU/SIC@C/NW samples was 0.74 and 0.76 vol. d. The peaks that characterized the proportion of polyurethane chains’ flexible and rigid segments that formed the boundary layer are painted in purple (2, 2′). The polymer’s total fraction forming the boundary layer (φ*_b_*) for the PU/SiC_neat and PU/SIC@C/NW samples was 0.17 and 0.14 vol. d. Yellow (*3*) is the peak that characterized the polymer’s fraction and formed a transitional (loose) layer that consisted mainly of adsorption loops that are not part of the boundary layer. The polymer’s total fraction forming the transition layer (φ*_t_*) for the PU/SiC_neat and PU/SiC@C/NW samples was 0.09 and 0.1 vol.d.

Table 3 shows the values of φ*_b_*, φ*_t_* and φ*_v_* obtained as a result of an approximation of the dependent G″(T) experimental curves, over the entire concentration range for PU/sic_neat and PU/SiC@C/NW samples.

### 3.3. The Boundary Layer Thickness Determination

The calculation of the boundary layer thickness is based on the idea of the thickness of the coating of a particle with a known surface area (*S_g_*), with a known volume of polymer consumed for the boundary layer’s formation (*V_pb_*). *V_pb_* is the product of the polymer in the composite’s (*V_p_*) total volume and φ*_b_* obtained from the peak areas’ sum (2, 2′). In article [32], to find the boundary layer thickness, considering the filler as a quasi-spherical particle with radius (*r*) and the number of filler particles per unit volume as *N* = (1 − *V_p_*)/(4/3) *Nr*^3^, the following formula was used:

*V_pb_* = ((4/3) π (*r* + δ)^3^ − (4/3) π*r*^3^)*N*, (2)

To minimize any numeric assumptions within the formula, we introduced the experimentally obtained value of the specific surface area, so the expression will take the form:

δ = (*V_p_*φ_b_)/(*S_g_m_f_*), (3)

where *V_p_* is the total volume of the polymer in the composite, φ*_b_* is the fraction of the polymer consumed for the boundary layer’s formation, *S_g_* is the specific surface area and *m_f_* is the mass of the filler in the composite.

**Figure 6:**
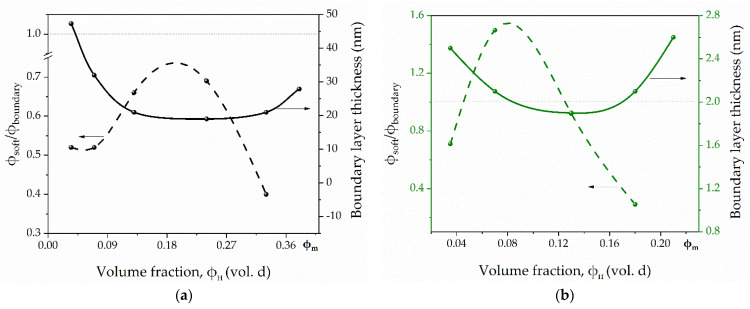
Dependence of φ*_t_*/φ*_b_* and δ on the volume fraction of the filler φ*_f_* in the samples PU/SiC_neat (**a**) and PU/SiC@C/NW (**b**).

Figure 6 shows the dependence of the boundary layer thickness and the ratio φ*_t_*/φ*_b_* on the filler’s volume fraction in the samples PU/SiC_neat and PU/SiC@C/NW.

As you can see from the graphs, the morphology of the particle surface does not affect the nature of the above dependencies. The boundary layer thickness in images filled with particles with both a smooth surface and a bristle-like relief has a nonlinear dependence on the filler fraction δ(φ*_f_*) and has a minimum in the area corresponding to 50% filling of the system. An increase in the specific surface area of filler particles leads to a decrease in the boundary layer thickness, its average value for PU/SiC_neat samples is 33 nm, and for PU/SiC@C/NW samples −2.25 nm. Furthermore, the dependence of the transition layer fraction ratio to the boundary layer’s fraction is characterized by nonlinearity. This indicates a complex process of the polymer matrix’s structure formation, filling it with a dispersed filler.

## 4. Conclusions

In conclusion, we have conducted a study of the features of the polyurethane matrix’s structure formation filling with dispersed SiC particles with different surface morphologies. On the basis of this, we can make following conclusions.

Modified SiC@C/NP and SiC@C/NW particles’ filling with a highly developed surface and a brush-like relief leads to a decrease in the range of variation in the *T_g_*(φ*_f_*) dependence.

An empirical method was proposed to find the boundary layer thickness and the proportion of polymer consumed for the formation of the boundary, transition and bulk layers of the polyurethane matrix. The boundary layer thickness calculations showed that the higher specific surface area of the modified filler particles SiC@C/NP and SiC@C/NW was due to the formation of a brush-like surface, and consequently, a thinner boundary layer was formed in the matrix structure. This reduced the degree of microheterogenicity of the polyurethane matrix’s structure in the composite and led to a decrease in the range of changes in the dependence of *T_g_*(φ*_f_*). In addition, it was found that the surface morphology did not affect the dependent’s nature δ(φ*_f_*), and had a minimum in the area corresponding to 50% filling of the system.

## Figures and Tables

**Table 1:** The maximum value of the filler’s volume fraction in the composite.

	SiC_neat	SiC@C	SiC@C/NP	SiC@C/NW
specific surface area (*S_g_*), m^2^ g^−l^	3	15	38.8	45
maximum packing volume fraction (φ*_m_*)	0.39	0.28	0.23	0.21

**Table 2:** Values of the weight concentration and volume filler’s fraction.

	Percent in Weight of Filler, wt. %								
	5	10	20	40	60	65	70	75	99
φf (SiC_neat)	0.02	0.035	0.08	0.15	0.19	0.21	0.22	0.28	**0.38**
φf (SiC@C)	0.02	0.035	0.08	0.15	0.19	0.21	0.22	**0.28**	
φf (SiC@C/NP)	0.02	0.035	0.08	0.15	0.19	0.21	**0.22**		
φf (SiC@C/NW)	0.02	0.035	0.08	0.15	0.19	**0.21**			

**Table 3:** The values of the polymer’s volume fractions, spending on the formation of the boundary, transition and volume layers of the matrix with the filler’s certain proportion in the composite.

φ*_f_*, vol.d	PU/SiC_neat			PU/SiC@C/NW		
	φ*_b_*, vol.d	φ*_t_*, vol.d	φ*_v_*, vol.d	φ*_b_*, vol.d	φ*_t_*, vol.d	φ*_v_*, vol.d
0.035	0.17	0.09	0.74	0.14	0.10	0.76
0.08	0.23	0.12	0.65	0.22	0.30	0.48
0.15	0.30	0.20	0.50	0.41	0.38	0.21
0.19	0.43	0.30	0.27	0.68	0.20	0.12
0.36	0.62	0.25	0.13			

## Data Availability

The data presented in this study are available on request from the corresponding author.
